# *Rhodoluna lacicola* gen. nov., sp. nov., a planktonic freshwater bacterium with stream-lined genome

**DOI:** 10.1099/ijs.0.065292-0

**Published:** 2014-09

**Authors:** Martin W. Hahn, Johanna Schmidt, Sami J. Taipale, W. Ford Doolittle, Ulrike Koll

**Affiliations:** 1 Research Institute for Limnology, University of Innsbruck, Mondseestrasse 9, A-5310 Mondsee, Austria; 2 Department of Biological and Environmental Science, University of Jyväskylä, PL 35 (YA), 40014 Jyväskylä, Finland; 3 Department of Biochemistry and Molecular Biology, Dalhousie University, Halifax, Nova Scotia, Canada

## Abstract

A pure culture of an actinobacterium previously described as ‘*Candidatus* Rhodoluna lacicola’ strain MWH-Ta8 was established and deposited in two public culture collections. Strain MWH-Ta8^T^ represents a free-living planktonic freshwater bacterium obtained from hypertrophic Meiliang Bay, Lake Taihu, PR China. The strain was characterized by phylogenetic and taxonomic investigations, as well as by determination of its complete genome sequence. Strain MWH-Ta8^T^ is noticeable due to its unusually low values of cell size (0.05 µm^3^), genome size (1.43 Mbp), and DNA G+C content (51.5 mol%). Phylogenetic analyses based on 16S rRNA gene and RpoB sequences suggested that strain MWH-Ta8^T^ is affiliated with the family *Microbacteriaceae* with *Pontimonas salivibrio* being its closest relative among the currently described species within this family. Strain MWH-Ta8^T^ and the type strain of *Pontimonas salivibrio* shared a 16S rRNA gene sequence similarity of 94.3 %. The cell-wall peptidoglycan of strain MWH-Ta8^T^ was of type B2β (B10), containing 2,4-diaminobutyric acid as the diamino acid. The predominant cellular fatty acids were anteiso-C_15 : 0_ (36.5 %), iso-C_16 : 0_ (16.5 %), iso-C_15 : 0_ (15.6 %) and iso-C_14 : 0_ (8.9 %), and the major (>10 %) menaquinones were MK-11 and MK-12. The major polar lipids were diphosphatidylglycerol, phosphatidylglycerol and two unknown glycolipids. The combined phylogenetic, phenotypic and chemotaxonomic data clearly suggest that strain MWH-Ta8^T^ represents a novel species of a new genus in the family *Microbacteriaceae*, for which the name *Rhodoluna lacicola* gen. nov., sp. nov. is proposed. The type strain of the type species is MWH-Ta8^T^ ( = DSM 23834^T^ = LMG 26932^T^).

Members of the phylum *Actinobacteria* frequently contribute large proportions to freshwater bacterioplankton of lakes, ponds and rivers (Glöckner *et al.*, 2000; Hahn, 2006; Newton *et al.*, 2011). Shares of 10–60 % of bacterioplankton cell numbers were frequently reported (Allgaier & Grossart, 2006; Glöckner *et al.*, 2000; Newton *et al.*, 2011; Salcher *et al.*, 2010); however, none of the abundant lineages of freshwater *Actinobacteria* is represented by a species with a validly published name (Newton *et al.*, 2011). Only eight *Candidatus* species representing six *Candidatus* genera have been described so far (Hahn, 2009; Jezbera *et al.*, 2009). Here we present the first species description of an abundant freshwater bacterium representing a lineage of indigenous freshwater bacteria in the phylum *Actinobacteria*.

Strain MWH-Ta8^T^ was obtained from the hypertrophic Meiliang (Mailing) Bay (31° 23′ 55″ N 120° 13′ 50″ E) Lake Taihu, People’s Republic of China (Hahn, 2009) by using the filtration-acclimatization method (Hahn *et al.*, 2004). Lake Taihu is a large and shallow subtropical lake characterized by a surface area of 2338 km^2^, a mean depth of 1.9 m and a maximum depth of only 2.6 m (Sun & Mao, 2008). The lake is the third largest freshwater lake in China and plays an important role as a freshwater source for a number of cities.

Initially only a mixed culture consisting of strain MWH-Ta8^T^ and uncharacterized members of the class *Betaproteobacteria* could be established, which only enabled the description of a *Candidatus* species, i.e. ‘*Candidatus* Rhodoluna lacicola’ MWH-Ta8 (Hahn, 2009). Further purification efforts finally resulted in establishment of a pure culture growing in liquid medium and on NSY agar plates. Strain MWH-Ta8^T^ shares with typical freshwater members of the phylum *Actinobacteria* characterized by fluorescent *in situ* hybridization (FISH) in water samples from freshwater systems, cell sizes of <0.1 µm^3^ (Salcher *et al.*, 2010). Bacteria with cell sizes below this threshold comprise the majority of cells in freshwater and marine bacterioplankton, and are termed ultramicrobacteria (UMB) (Duda *et al.*, 2012). Interestingly, strain MWH-Ta8^T^ represents an obligate UMB (Duda *et al.*, 2012), which maintains its small cell sizes even when grown in rich complex media.

Strain MWH-Ta8^T^ strongly differed in cell size from all previously described species of the family *Microbacteriaceae*, and most likely differed from them all in growth potential when cultivated and tested by using standard bacteriological methods. Previous experiments on phenotypic characterization of various freshwater actinobacteria by using API 50 CH tests (bioMérieux) did not result in any positive response; therefore, a non-standard test method for subfstrate utilization (see below) previously used for other strains with only weak growth potential was used for the characterization of strain MWH-Ta8^T^. Another problem was encountered when we tried to evaluate the results of Gram staining tests by microscopy at a magnification of ×1000. Due to the tininess of the stained cells, it was impossible to recognize any colours, which made it impossible to conclude which Gram stain reaction occurs. Several of the performed phenotypic tests (see below) resulted only in weak reactions that were difficult to interpret. Repetition of such experiments often did not improve the quality of the test result. One consequence of the unusual traits of strain MWH-Ta8^T^ is that it was not possible to fulfil the minimal standards for the description of new taxa of the suborder *Micrococcineae* (Schumann *et al.*, 2009). We compensated for the limited phenotypic characterization of strain MWH-Ta8^T^ by a characterization of the genome of this strain and by an extended phylogenetic analysis.

## 

### Phylogeny.

The 16S rRNA gene sequence of strain MWH-Ta8^T^ used for phylogenetic analyses was established as described previously (Hahn, 2009). This sequence was obtained from the previous mixed culture; however, the sequence is identical to the 16S rRNA gene sequence determined by genome sequencing (see below) of the later established pure culture of the strain. For phylogenetic inference, aligned ribosomal sequences of reference strains were downloaded from the Greengenes database (DeSantis *et al.*, 2006), while sequences not available from this source were retrieved from the GenBank database. The latter sequences were aligned by using already aligned sequences as a template, and the whole alignment was examined for errors, which were then manually corrected. The final alignment consisted of 1405 positions, and the length of the aligned sequence of strain MWH-Ta8^T^ was 1365 bp (without alignment gaps). Maximum-likelihood (ML), neighbour-joining (NJ) and maximum-parsimony (MP) trees were calculated with the mega software version 5.2 (Tamura *et al.*, 2011). The best substitution model for phylogeny inference by the ML method was searched by the model test option of mega 5.2. Accordingly, the General Time Reversible model with non-uniformity of evolutionary rates among sites by using a discrete Gamma distribution (+G) with five rate categories and by assuming that a certain fraction of sites are evolutionarily invariable (+I) was used for calculation of a ML tree. A NJ tree was calculated by using the Tamura-3-parameter model with a gamma distribution with five categories, a heterogeneous pattern among lineages, and pairwise deletion of gaps. All alignment positions (all sites) were used in the calculation of a MP tree. The number of bootstrap replications in calculation of the ML, NJ and MP trees was 1000, 1000 and 100, respectively. In all trees based on a 16S rRNA gene sequence set consisting of the type species of all described genera within the family *Microbacteriaceae* and some closely related *Candidatus* species, the sequence of strain MWH-Ta8^T^ clustered within this family (Fig. 1). As already revealed by previous analyses, strain MWH-Ta8^T^ formed, together with other freshwater actinobacteria, a tight cluster (Hahn, 2009) previously designated Luna-1 cluster (Hahn *et al.*, 2003). This cluster was supported by all three phylogenetic methods (Fig. 1). This freshwater cluster including strain MWH-Ta8^T^ appeared in all trees calculated by the three methods together with *Pontimonas salivibrio* CL-TW6^T^ (Jang *et al.*, 2013). The close relationship of these taxa was supported with high bootstrap values by at least two methods, but the phylogenetic relationship of the *Pontimonas*/Luna-1 cluster to other genera of the family *Microbacteriaceae* could not be resolved. The *Pontimonas*/Luna-1 cluster appeared in all three trees together with the type species of six other genera, but this relationship was not supported by high bootstrap values in any tree, and the branching order of these taxa markedly differed between trees calculated by using different methods. Furthermore, *Pontimonas salivibrio* clustered in the trees presented here (Fig. 1) with other taxa than in a previous phylogenetic reconstruction (Jang *et al.*, 2013).

**Fig. 1.**
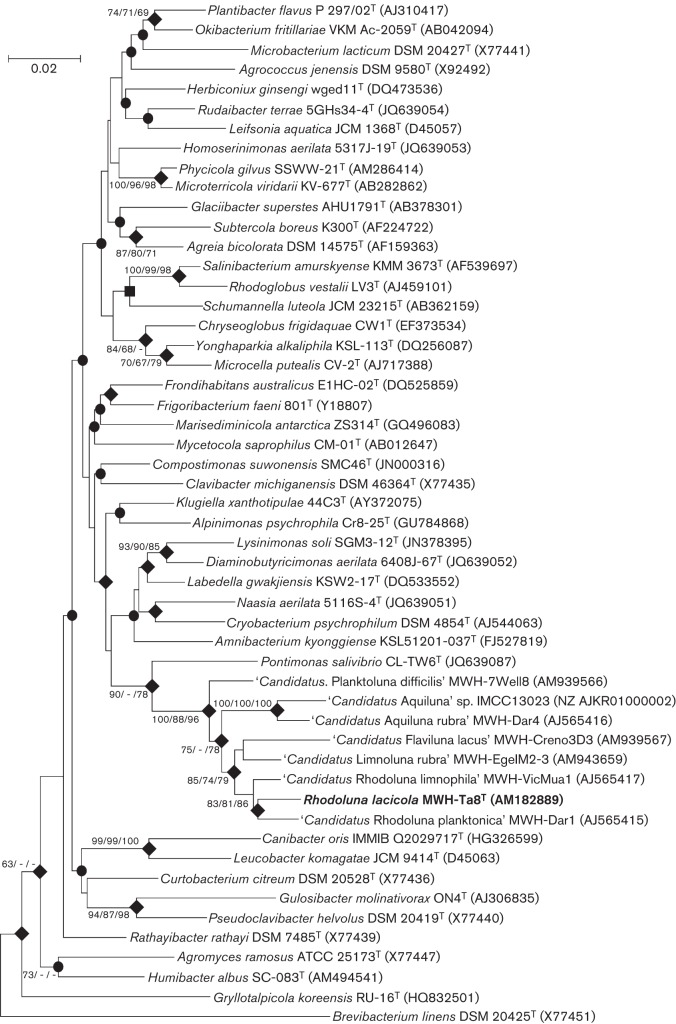
NJ tree based on partial 16S rRNA gene sequences reconstructing the phylogenetic position of strain MWH-Ta8^T^ within the family *Microbacteriaceae*. Results from analyses by the ML and MP methods are also indicated. Bootstrap values (percentage of replicates) above the threshold of ≥60 % are shown for those nodes supported in at least one of the three methods; these bootstrap values are depicted in the order NJ/ML/MP. Filled diamonds indicate nodes reconstructed by all three methods independent of their respective bootstrap values; filled circles indicate those nodes only present in NJ and ML trees; filled square indicates one node only reconstructed by the NJ and MP methods. *Brevibacterium linens* DSM 20425^T^, which is not affiliated with the family *Microbacteriaceae* was used as an out-group. GenBank accession numbers of 16S rRNA gene sequences are depicted in parentheses. Bar, 0.02 substitutions per nucleotide position.

Because strain MWH-Ta8^T^ greatly differed in a couple of important taxonomic traits from all previously described taxa affiliated with the family *Microbacteriaceae*, we additionally evaluated the phylogenetic position of the strain by using amino acid (aa) sequences of the beta subunit of the RNA polymerase (RpoB) retrieved from genome databases. Forty-three genomes of strains classified as *Microbacteriaceae* were found in the integrated microbial genomes (IMG) database (Markowitz *et al.*, 2012) and one additional genome assigned to this family but not contained in the IMG database was found in the GenBank database. Because only 20 of those 44 genomes represented taxonomically evaluated type species (Table S1, available in the online Supplementary Material), their putative affiliation with the family *Microbacteriaceae* was first checked by a phylogenetic analysis of their 16S rRNA gene sequences. Tree reconstruction and other analyses confirmed for 42 strains, a putative affiliation with the family *Microbacteriaceae* (Fig. S1). One strain (*Microbacterium* sp. KROCY2) clustered outside the family *Microbacteriaceae*, and one strain could not be tested because its draft genome completely lacked a gene annotated as 16S or SSU rRNA gene (Table S1).

Nucleotide sequences of the *rpoB* gene of reference strains were retrieved, translated to aa sequences and aligned by the muscle aligner provided by the mega software package. The complete RpoB aa sequence of strain MWH-Ta8^T^ had a length of 1204 aa, while 1103 aa were contained in the aligned and trimmed sequence set used for the phylogenetic analyses. NJ, ML and MP trees were reconstructed by using mega software version 6.05. The ML tree was calculated, as suggested by model test, by using the LG model +G +I. The NJ tree was calculated by using the Poisson model and a gamma distribution (+G) with a parameter of five. For calculation of ML, NJ and MP trees all alignment columns containing gaps were completely omitted, and 1000, 1000 and 100 bootstrap replications were performed in ML, NJ and MP analyses, respectively. The obtained RpoB trees support the affiliation of strain MWH-Ta8^T^ with the family *Microbacteriaceae* (Fig. 2) but in contrast to the phylogenetic reconstruction based on 16S rRNA gene sequences (Fig. 1), strain MWH-Ta8^T^ did not appear in a nested position within the family. The difference in position within the family could have resulted from the different sets of taxa used in the two phylogenetic reconstructions. Interestingly, strain MWH-Ta8^T^ clustered together with ‘*Candidatus* Aquiluna’ sp. IMCC13023 in both the 16S rRNA gene tree and the RpoB tree, but the relationship to *Pontimonas salivibrio* and the type species of six other genera appearing close to strain MWH-Ta8^T^ in the 16S rRNA gene trees could not be evaluated since RpoB sequences of these taxa were not available.

**Fig. 2.**
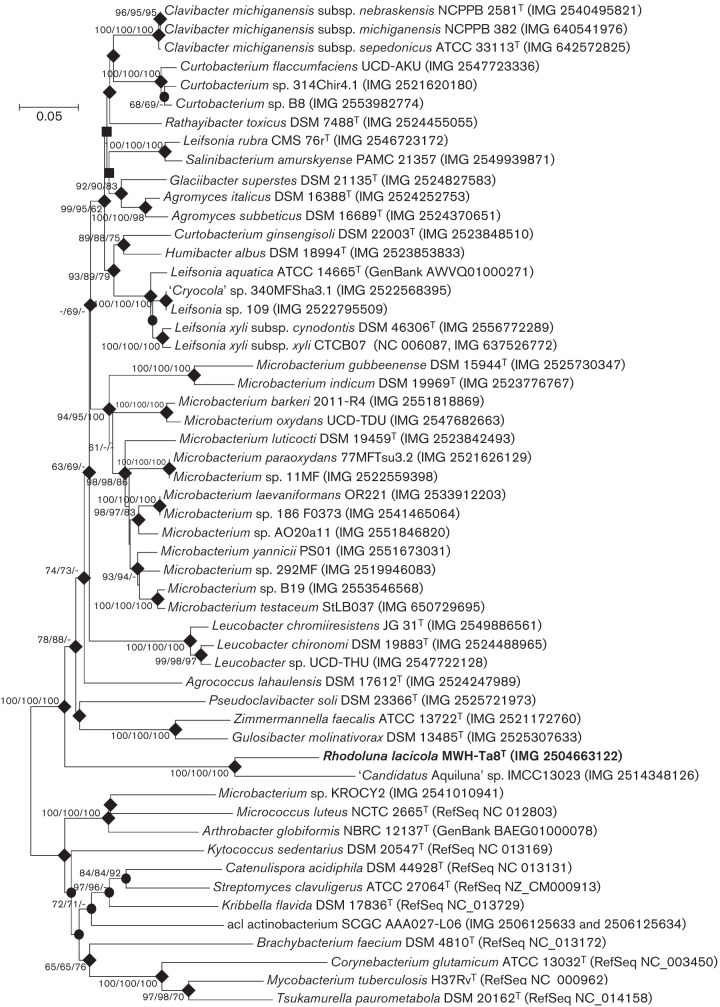
NJ tree based on partial RpoB (DNA-directed RNA polymerase subunit beta) amino acid sequences reconstructing the phylogenetic position of strain MWH-Ta8^T^. Amino acid sequences were used for phylogenetic analysis in order to omit phylogenetic noise potentially cause by the pronounced differences in DNA G+C content of *rpoB* genes (range 51 to 68 mol%). Results from analyses by the ML and MP methods are also indicated. Bootstrap values (percentage of replicates) above the threshold of ≥60 % are shown for those nodes supported at least in inference by one of the three methods; values are presented in the order NJ/ML/MP. Filled diamonds indicate nodes reconstructed by all three methods independent of their respective bootstrap values; filled circles indicate those nodes only present in NJ and ML trees; filled squares indicate nodes only reconstructed by the NJ and MP methods. If available, IMG Gene ID numbers of genes encoding the RpoB proteins are given in parentheses; for other proteins, the RefSeq or GenBank accession numbers are provided. *Microbacterium* sp. KROCY2 represents a strain classified by the IMG database as a member of the family *Microbacteriaceae*, and acI actinobacteria SCGC AAA027-L06 represents a freshwater actinobacterium affiliated with the acI lineage (Garcia *et al.*, 2013). Ten type strains of species not affiliated with the family *Microbacteriaceae* were used as an out-group. Bar, 0.05 substitutions per amino acid position.

16S rRNA gene sequence similarities were calculated by using the mega software. Based on the sequence alignment, a matrix of pairwise nucleotide differences was calculated, and the numbers of nucleotide differences were converted to pairwise sequence similarity values. Strain MWH-Ta8^T^ did not share sequence similarity values >97 % with any of the 43 type species of the currently described genera affiliated with the family *Microbacteriaceae*. The highest similarities were observed with sequences of *Alpinimonas psychrophila* Cr8-25^T^ (94.5 %), *Pontimonas salivibrio* CL-TW6^T^ (94.3 %) and *Compostimonas suwonensis* SMC46^T^ (94.2 %).

### Phenotypic and chemotaxonomic traits.

Strain MWH-Ta8^T^ formed small, circular, convex colonies with a shiny surface and a red pigmentation on NSY agar plates (Hahn, 2009); however, unpigmented mutants were also observed and one such strain (MWH-Ta8n) was deposited at the BCCM/LMG culture collection under the accession number LMG 27024. Cell morphology and size (Table 1) were examined by epifluorescence microscopy (Axioplan, Zeiss, Germany) after staining with the fluorochrome 4′,6-diamidino-2-phenylindole dihydrochloride (DAPI) at a magnification of ×1250 as described previously (Hahn *et al.*, 2003). This method enabled a suitable microscopic examination of this ultramicrobacterium. Due to tininess of cells, observation of motility by phase-contrast microscopy (×1000) did not yield reliable results. Therefore, tests by culturing strain MWH-Ta8^T^ on soft agar (NSY medium, 0.4 % agar) were performed. Repeated experiments suggested a weak potential for spreading on the agar surface; however, the absence of genes required for flagellum synthesis in the genome sequence of the strain (see below) suggested that the strain was negative for motility by flagella (Table 1).

**Table 1.** Morphological and phenotypic traits that characterize strain MWH-Ta8^T^ −, Negative; +, positive; w, weakly positive.

Characteristic	MWH-Ta8^T^
Cell morphology	Curved (selenoid) rods
Cell length (µm)	0.85
Cell width (µm)	0.30
Cell volume (µm^3^)	0.053
Pigmentation	Red
Motility	−
Temperature range of growth (°C)	9–36 (w at 37 °C)
NaCl tolerance (%, w/v)	0–0.4 (w at 0.5 %)
Anaerobic growth	−
Catalase	**+**
Oxidase	−
Assimilation of:	
Formic acid	−
Glyoxylic acid	−
Glycolic acid	−
Propionic acid	−
Acetic acid	w
Fumaric acid	−
Malonic acid	−
Oxaloacetic acid	w
Citric acid	−
d-Fucose	−
d-Glucose	−
d-Mannose	**+**
d-Sorbitol	**+**
l-Glutamate	w
l-Alanine	w
Betaine	−

The temperature range of growth and salinity tolerance were tested by cultivation on NSY agar plates (Hahn *et al.*, 2004). Inocula from cultures grown at room temperature in liquid NSY medium were spread on standard NSY plates and incubated at different temperatures, or were spread on NSY plates supplemented with 0, 0.05, 0.1, 0.2, 0.3, 0.4, 0.5, 0.6, 0.7, 1.0, 1.5 or 2.0 % (w/v) NaCl and incubated at room temperature. Temperature experiments were performed at 4, 8, 9, 15, 17, 25, 30, 34, 36, 37 and 39 °C. These experiments suggested a mesophilic thermal adaptation and a low salinity tolerance (Table 1). Growth under anoxic conditions on standard NSY agar and NSY agar supplemented with nitrate (0.8 mM) was examined by using an anaerobic chamber [anoxic conditions generated by Anerocult A (Merck)]. Catalase activity was determined by bubble production in 3 % (v/v) H_2_O_2_, and cytochrome oxidase activity was examined by using Bactident Oxidase test stripes (Merck). The latter test was repeated several times and resulted in comparison to oxidase-positive reference strains only in very faint bluish reactions, which were evaluated as negative test results. Due to difficulties in the examination of substrate utilization of strain MWH-Ta8^T^ by standard methods, a method previously successfully applied for characterization of novel members of the genus *Polynucleobacter* (Hahn *et al.*, 2009) was employed. Briefly, growth enabled by utilization of a specific substrate was determined by comparison of the optical density established in liquid one-tenth-strength NSY medium (0.3 g) with and without 0.5 g test substance. OD 575 differences of 10 %, 10–50 % and 50 % of the OD 575 established in the medium without test substance were scored after 10 days of growth as no utilization, weak utilization and good utilization, respectively. Good utilization was only observed for d-mannose and d-sorbitol (Table 1).

The analyses of peptidoglycan structure, respiratory quinones and polar lipids were carried out by the Identification Service of the Leibniz-Institut DSMZ (Deutsche Sammlung von Mikroorganismen und Zellkulturen), Braunschweig, Germany. For these analyses and for fatty acid analysis, biomass of strain MWH-Ta8^T^ was harvested by centrifugation from cultures grown in liquid NSY medium on a shaker (50 r.p.m.) at 22–24 °C. Peptidoglycan analyses were performed as described previously (Schumann, 2011; Schumann *et al.*, 2012). The total hydrolysate (4 M HCl, 100 °C, 16 h) of the peptidoglycan of strain MWH-Ta8^T^ contained the amino acids 2,4-diaminobutyric acid (Dab), alanine (Ala), glycine (Gly), homoserine (Hse) and glutamic acid (Glu) in an approximate molar ratio of 0.8 : 0.5 : 0.9 : 0.4 : 1.0, respectively. The partial hydrolysate (4 M HCl, 100 °C, 0.75 h) contained the peptides Mur–Gly, d-Ala–d-Dab, Gly–d-Glu. The latter peptide is characteristic of B-type peptidoglycan, and it was concluded that the peptidoglycan of strain MWH-Ta8^T^ represented the type B2β (type B10) (Schleifer & Kandler, 1972; Schumann, 2011). The respiratory quinones of strain MWH-Ta8^T^ were menaquinones MK-11 (56 %), MK-12 (24 %), MK-10 (9 %), MK-9 (5 %), MK-13 (2 %) and some minor components. The major polar lipids were diphosphatidylglycerol, phosphatidylglycerol and two unknown glycolipids.

Cellular fatty acids from whole bacteria samples were extracted with a 4 : 2 : 1 (by vol.) chloroform/methanol/water mixture (Parrish, 1999). These samples were then sonicated and vortexed twice and the organic phases were removed and pooled. Fatty acids were transmethylated at 50 °C overnight using 1 % sulfuric acid as a catalyst. Fatty acid methyl esters were analysed with a gas chromatograph (Agilent 6890N) with mass spectrometric detection (Agilent 5973N). An Agilent DB-23 column (30 m×0.25 mm×0.15 µm) was used with the following temperature program: 60 °C was maintained for 1.5 min, then the temperature was increased at 10 °C min^−1^ to 100 °C, followed by 2 °C min^−1^ to 140 °C, and 1 °C min^−1^ to 180 °C, and finally heated up at 2 °C min^−1^ to 210 °C and then held for 6 min. Helium was used as carrier gas with a mean velocity of 34 cm s^−1^. The predominant cellular fatty acids of strain MWH-Ta8^T^ were anteiso-C_15 : 0_, iso-C_16 : 0_, iso-C_15 : 0_ and iso-C_14 : 0_ (Table 2). Strain MWH-Ta8^T^ differed only slightly in cellular fatty acid composition from the phylogenetically most closely related characterized species, *Pontimonas salivibrio* CL-TW6^T^.

**Table 2.** Comparison of cellular fatty acids contents of strain MWH-Ta8^T^ and the phylogenetically most closely related strain *Pontimonas salivibrio* CL-TW6^T^ tr, Traces; nd, not detected.

Fatty acid	MWH-Ta8^T^	*Pontimonas salivibrio* CL-TW6^T^
Saturated		
C_14 : 0_	2.2	2.2
C_15 : 0_	1.4	2.6
C_16 : 0_	7.0	7.0
C_17 : 0_	tr	tr
C_18 : 0_	2.5	1.1
Total saturated	13.1	12.9
Branched		
iso-C_14 : 0_	8.9	11.8
iso-C_15 : 0_	15.6	13.2
anteiso-C_15 : 0_	36.5	32.6
iso-C_16 : 0_	16.5	20.4
iso-C_17 : 0_	2.4	1.9
anteiso-C_17 : 0_	4.1	2.8
Total branched	75.1	70.9
Monounsaturated		
C_16 : 1_ω7	nd	nd
C_18 : 1_ω7	tr	tr
Total monounsaturated	0.0	0.0

### Genomic traits.

DNA used for genome sequencing was extracted from biomass of strain MWH-Ta8^T^ grown in liquid NSY medium. The extraction was performed as described previously (Meincke *et al.*, 2012). Construction of a paired-end library and sequencing by using a Roche GS FLX system and Titanium chemistry was performed at McGill University and Génome Québec Innovation Centre (Montréal, Canada). The paired-end library had an mean insert length of approximately 7000 kb, and sequencing of the library resulted in 239 000 filtered reads with median length of 323 bp. Sequence assembly by using the GS *De novo* Assembler software (Newbler software, Roche) resulted in four large contigs, which were ordered and oriented into one scaffold. Gap closure was performed by primer design, PCR amplification and Sanger sequencing of obtained amplicons. The closed genome consists of 1 430 433 bp with a G+C content of 51.53 mol%. The closed genome sequence was annotated by using the IMG/ER annotation pipeline (Markowitz *et al.*, 2012). The genome of strain MWH-Ta8^T^ differed strongly in genome size and G+C content from the vast majority of genome sequences of members of the phylum *Actinobacteria* (1296 genomes; excluding draft genomes obtained from single cells) currently (February 2014) available in the IMG database (Markowitz *et al.*, 2012) (Fig. 3). Only five of the 1296 sequences possessed smaller genome sizes than strain MWH-Ta8^T^. Four of these five genomes represent obligately host-associated genera of the phylum *Actinobacteria* (*Tropheryma*, *Atopobium* and ‘*Candidatus* Ancillula’), while the only free-living strain with a smaller genome size was ‘*Ca.* Aquiluna’ sp. IMCC13023 (Kang *et al.*, 2012), which represented a close relative of strain MWH-Ta8^T^ (Figs 1 and 2). A very small genome size of about 1.2 Mbp was suggested recently for a member of the so-called acI lineage of freshwater actinobacteria (Garcia *et al.*, 2013). This estimation of genome size is based on a draft genome sequence obtained from a single cell and consists of 75 contigs. The phylogenetic position and taxonomic affiliation of the acI lineage, which is represented by ‘*Candidatus* Planktophila limnetica’, could not be determined with certainty so far (Jezbera *et al.*, 2009); however, it is obvious that this lineage of freshwater *Actinobacteria* has a different phylogenetic origin than strain MWH-Ta8^T^ (Fig. 2).

**Fig. 3.**
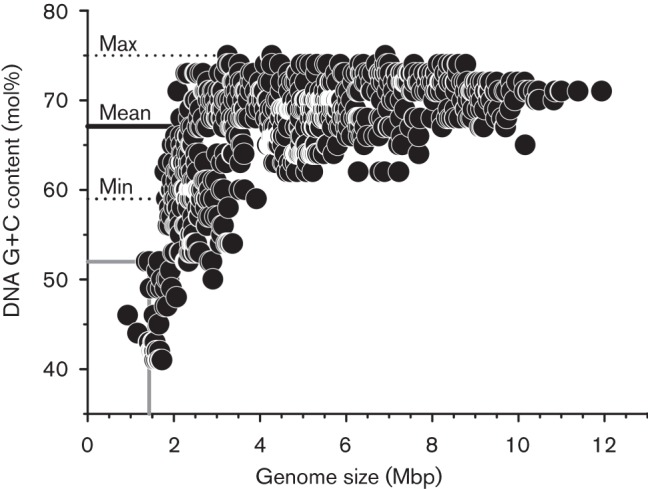
Genome size and DNA G+C content of all genomes of actinobacteria currently contained in the IMG database. Only genomes of strains established as pure cultures were considered. Values of strain MWH-Ta8^T^ are indicated by solid grey lines, and minimum, mean and maximum DNA G+C values of type strains of members of the family *Microbacteriaceae* (Table S1) are indicated by dotted and solid black lines.

Apart from the genomes of strain MWH-Ta8^T^ and ‘*Ca.* Aquiluna’, the DNA G+C content of genomes putatively affiliated with the family *Microbacteriaceae* (Table S1) ranged from 59 to 73 mol% (mean 68.9 mol%; *n* = 42). These data are quite similar to the DNA G+C content of type strains of previously described species of the family *Microbacteriaceae* (Table 3). Values for these type strains ranged from 59 mol% [*Alpinimonas* (Schumann *et al.*, 2012) and *Schumannella* (An *et al.*, 2008)] to 76 mol% [*Agrococcus* (Zhang *et al.*, 2010)] and the mean DNA G+C content was 67.1 mol%. Thus, strains MWH-Ta8^T^ and ‘*Ca.* Aquiluna’ sp. IMCC13023 strongly differ in both traits, genome size and DNA G+C content, from previously described species of the family *Microbacteriaceae*.

**Table 3.** Genome characteristics of strain MWH-Ta8^T^ and other strains affiliated with the family *Microbacteriaceae* The column presenting data for other members of the family *Microbacteriaceae* summarizes in the first three lines characteristics of 41 genome-sequenced strains (Table S1) and in the last line data of type species determined by a different method. Phylogenetic analyses with 16S rRNA gene sequences of the genome-sequenced strains confirmed their affiliation with the family *Microbacteriaceae* (Fig. S1). The data presented in the last column represent minimum–maximum (mean) values. The genome sequence of MWH-Ta8^T^ represents a closed genome organized in one contig, while the genome sequence strain IMCC13023 is organized in six scaffolds (Kang *et al.*, 2012). nd, Not determined.

Characteristic	MWH-Ta8^T^	‘*Ca.* Aquiluna’ sp. IMCC13023	Other *Microbacteriaceae*
Genome Size (Mbp)	1.43	1.36	2.2–4.8 (3.3)
Putative ORFs	1408	1410	2110–4766 (3298)
DNA G+C content (mol%)*	51.5	51.7	59–73 (68.9)
DNA G+C content (mol%)†	nd	nd	59–76 (67.1)

* Determined by genome sequencing.

† Determined by HPLC analyses.

The genome of strain MWH-Ta8^T^ encoded a putative phosphotransferase system (PTS) for fructose but a PTS for glucose was lacking. A complete gene set for gluconeogenesis was found but a gene encoding an enzyme for phosphorylation of fructose 6-phosphate to fructose 1,6-bisphosphate [e.g. a gene for phosphofructokinase-1 (EC 2.7.1.11)], which represents a crucial step of the glycolysis pathway, was missing in the genome annotations. A complete citrate cycle is encoded and all genes required for a respiratory electron transport chain were present in the annotated genome. On the other hand, the genome lacked genes encoding a cytochrome *bd* complex, which suggests that the strain is not adapted to low oxygen concentrations. Putative ABC transporters for phosphate, iron (III) ions, spermidine/putrescine, sorbitol/mannitol, d-xylose, and branched-chain amino acids were found in the genome. Two ammonium transporter genes of the *amt* family and genes encoding ammonia assimilation were found. Assimilatory and respiratory nitrite and nitrate reductases were lacking, which suggested that strain MWH-Ta8^T^ is able to assimilate ammonia but not nitrate or nitrite. Furthermore, the genome of the strain completely lacked genes of flagellum synthesis.

The genome of strain MWH-Ta8^T^ also encoded a putative actinorhodopsin/proteorhodopsin (Sharma *et al.*, 2009). These proteins represent putative light-driven proton pumps enabling conservation of light energy. Only two of the 42 investigated reference genomes of members of the family *Microbacteriaceae* also encoded such genes. One of these two genomes (‘*Ca.* Aquiluna’ sp. IMCC13023) is also affiliated with the Luna-1 cluster of freshwater actinobacteria (Figs 1 and 2). As demonstrated previously (Sharma *et al.*, 2009) the majority of investigated members of this cluster encoded actinorhodopsin genes. Interestingly, the second strain among the reference genome strains that possessed such a gene, *Leifsonia rubra* CMS 76r^T^, was also isolated from an aquatic habitat, i.e. from a cyanobacterial mat of a pond in McMurdo dry valleys, Antarctica (Kumar Pinnaka *et al.*, 2013).

The genome of strain MWH-Ta8^T^ represented a strongly streamlined genome sharing an equally small genome size with ‘*Candidatus* Pelagibacter ubique’ (Giovannoni *et al.*, 2005b) a member of the marine SAR11 cluster (*Alphaproteobacteria*). Interestingly, both organisms also share the presence of proteorhodopsin/actinorhodopsin genes (Giovannoni *et al.*, 2005a), but they differ strongly in their ability to grow in artificial media at high substrate concentrations or on the surface of agar plates (Carini *et al.*, 2013).

### Ecology and biogeography.

Strain MWH-Ta8^T^ was isolated from the water column of a freshwater lake, suggesting that the strain possessed a planktonic lifestyle. This assumption is supported by the revealed genome characteristics, which are similar to those observed for other planktonic strains (Giovannoni *et al.*, 2005b), as well as by detection of closely related ribotypes, which shared >99 % 16S rRNA gene sequence similarity and the presence of two diagnostic sequences defining ‘*Ca.* Rhodoluna lacicola’ (Hahn, 2009) with strain MWH-Ta8^T^, in the water column of various freshwater systems. Three related strains were obtained as mixed cultures from freshwater systems located in Australia (GenBank accession number AM999979), China (AM999980) and Austria (FJ545223). These bacteria also share with strain MWH-Ta8^T^ small cell sizes and red pigmentation. In total, nine ribosomal sequences retrieved by cultivation-independent methods, which also share high sequence similarity and the presence of the two diagnostic sequences, originate from freshwater and estuary systems located in North America (HQ530784, EU800868, AY947900, AY947943, AF289150 and EF471688) and lakes located in China (JN232906, JF697478 and JF697420). These data indicate that the narrow phylogenetic taxon including strain MWH-Ta8^T^ consists of planktonic freshwater bacteria with a worldwide distribution, at least in freshwater systems of the temperate and subtropical climate zone.

### Proposal of a novel genus and species.

The results on phylogeny, phenotypic and chemotaxonomic characteristics of strain MWH-Ta8^T^ suggest that this strain represents a novel species of a new genus in the family *Microbacteriaceae*. This strain can be well distinguished from *Pontimonas salivibrio* strain CL-TW6^T^ (Table 4), which represented the closest related described species. In fact, strain MWH-Ta8^T^ can be easily distinguished from all previously described species of the family due to its unusually small cell size and the unusually low DNA G+C content (Table 3). According to the previous description of strain MWH-Ta8^T^ as ‘*Ca.* Rhodoluna lacicola’, we propose for this new taxon the name *Rhodoluna lacicola* gen. nov., sp. nov.

**Table 4.** Selected differential characteristics of strain MWH-Ta8^T^ and the type strain of the most closely related described species

Characteristic	MWH-Ta8^T^	*Pontimonas salivibrio* CL-TW6^T^*
Isolation source	Freshwater, lake	Seawater, solar saltern
Mean cell length >1 µm	−	+
Growth at <15 °C	+	−
Salt tolerance (%)	0–0.6	1–9
Utilization of d-glucose	−	+
Major respiratory quinones (MK)	11, 12	9, 10
Homoserine present in peptidoglycan	+	−
DNA G+C content (mol%)	51.5	60.0

* Data from Jang *et al.* (2013).

## Description of *Rhodoluna* gen. nov.

*Rhodoluna* [Rho.do.lu′na. Gr. n. *rhodon* the rose; L. fem. n. *luna* the moon; N.L. fem. n. *Rhodoluna* red-coloured moon, referring to the red pigmentation and the seemingly selenoid (crescent shape) morphology of the strain].

Aerobic chemo-organoheterotrophs. Cells are tiny, curved (selenoid) rods with cell volumes <0.1 µm^3^. The predominant cellular fatty acids are anteiso-C_15 : 0_, iso-C_16 : 0_, iso-C_15 : 0_ and iso-C_14 : 0_. The polar lipid profile contains diphosphatidylglycerol, phosphatidylglycerol and unknown glycolipids. The major menaquinones are MK-11 and MK12. The cell-wall peptidoglycan is of the B-type and contains 2,4-diaminobutyric acid as the diamino acid. The DNA G+C content is circa 52 mol%. The genome size is circa 1.4 Mbp. The type species is *Rhodoluna lacicola*.

## Description of *Rhodoluna lacicola* sp. nov.

*Rhodoluna lacicola* [la.ci′co.la. L. masc. n. *lacus* lake; L. suffix n. -*cola* (from *incola* the inhabitant); N.L. masc. n. *lacicola* inhabitant of lakes].

General descriptions of morphological and chemotaxonomic features are as given in the genus description. Cells grown in liquid NSY medium are tiny, curved (selenoid) rods with a size of about 0.3×0.9 µm and cell volume of 0.04–0.06 µm^3^. Colonies on NSY agar are small, circular and convex with a shiny surface and red pigmentation. Unpigmented mutants may occur. Grows on NSY medium aerobically but not under anaerobic conditions. The temperature range for growth on NSY is 9 to 36 °C, and weak growth may occur at 37 °C. NaCl concentrations of 0–0.4 % in NSY medium are well tolerated. Weak growth may occur at concentrations of 0.5 % NaCl but growth is absent at higher salinities. Positive result in tests for catalase activity, but only very weak signals in tests for oxidase activity (scored as oxidase-negative). Assimilates d-mannose and d-sorbitol, but not d-glucose, d-fucose, propionic acid, fumaric acid, malonic acid, citric acid or betaine. Lacks genes for flagella synthesis, phosphofructokinase and cytochrome *bd* complex, but encodes an actinorhodopsin gene (GenBank accession no. FJ545221). Furthermore, characterized by the combined presence of two oligonucleotide sequences, 5′-CTTGCTCCGGTGGATTAGTGG-3′ (*Escherichia coli* positions 83–105) and 5′-ACGACACCTTGGGGCATCCCAGGGTGTGGAA-3′ (*E. coli* positions 181–196), within the 16S rRNA gene.

The type strain, MWH-Ta8^T^ ( = DSM 23834^T^ = LMG 26932^T^), was isolated from the water column of hypertrophic Mailing Bay, Lake Taihu, China. The G+C content of the genomic DNA of the type strain is 51.5 mol% and the genome size is 1.43 Mbp.
